# The Ventilatory Changes of Pediatric Peroral Endoscopic Myotomy Patients

**DOI:** 10.4274/TJAR.2024.241609

**Published:** 2024-07-12

**Authors:** Mete Manici, Agah Rauf İşgüzar, Umut Deniz Adanur, Yavuz Gürkan, Muhammed Selman Söğüt, Fatih Aslan, Çiğdem Arıkan

**Affiliations:** 1 Koç University Hospital, Clinic of Anaesthesiology and Reanimation, İstanbul, Turkey; 2 University Hospital of Derby and Bourton, Department of Anaesthesiology and Reanimation, Derby, United Kingdom; 3 Koç University Hospital, Clinic of Gasroenterology and Hepatology, İstanbul, Turkey; 4 Koç University Hospital, Clinic of Pediatrics, İstanbul, Turkey

**Keywords:** Anaesthesia complications, general anaesthesia, peroral endoscopic myotomy

## Abstract

**Objective:**

Peroral endoscopic myotomy (POEM) has proven to be a successful treatment method for achalasia in both adult and pediatric patients. Yet, there is a lack of evidence for anaesthetic management of pediatric patients who underwent POEM procedure. In this study, we aim to present perioperative and postoperative management strategies for pediatric patients with achalasia from in anaesthesia aspect.

**Methods:**

Medical records were reviewed for 16 pediatric patients at a single center who underwent POEM procedure for achalasia between 2017 and 2020. Patients’ data regarding demographics, preoperative diet, body mass index, perioperative monitoring and vitals, airway management, anaesthesia maintenance, mechanical ventilation settings duration of recovery, length of stay, pain management and adverse events were evaluated.

**Results:**

The study cohort included 7 female and 9 male patients with a mean age of 5.5 years. Anaesthesia maintenance was provided with 0.8-1.2 minimum alveolar concentration sevoflurane in a 40-60% O2-air mixture, Remifentanil infusion and bolus doses of Rocuronium. The median age was 3 years for patients ventilated in pressure controlled ventilation mode and 10 years in volume controlled ventilation mode. Respiration rate and minute ventilation were adjusted to maintain end tidal carbon dioxide (ETCO2) below 45 mmHg. Needle decompression was applied for 14 patients (87.5%) for treatment of capnoperitoneum. The mean procedure duration and recovery room duration were 66 (±22.9) minutes and 62 (±21) minutes, respectively. Postoperative pain management is provided with paracetamol and tramadol in total 8 patients (50%). There was no adverse event during postoperative period and all patients discharged in a mean time of 3 days.

**Conclusion:**

POEM has demonstrated encouraging outcomes in terms of safety and effectiveness in pediatric patients. Due to challenging nature of the pediatric patients, it is important to acknowledge that the procedure requires specialized anaesthesia management. Management of perioperative complications of increased ETCO2 requires understanding the physiologic results of pneumo-mediastinum and pneumo-peritoneum. Beside the known anaesthetic management strategies, a tailored approach should be adopted for each patient. Further investigations should be conducted to develop standardized management.

**Main Points**

⦁ Peroral endoscopic myotomy in pediatric patients.

⦁ Peak airway pressures.

⦁ Pneumo-mediastinum and pneumo-peritoneum.

## Introduction

Achalasia is a motility disorder of the esophagus, characterized by inadequate relaxation of the lower esophageal sphincter (LES) and loss of peristalsis. It manifests with vomiting, regurgitation, recurrent cough, chest pain and weight loss in children. Untreated achalasia might lead to serious complications like megaesophagus, aspiration pneumonia and esophageal rupture. Treatment options include endoscopic pneumatic balloon dilation, Botulinum toxin injection and Heller myotomy.^1^ Treatment of children with achalasia has evolved in recent years with the introduction of peroral endoscopic myotomy (POEM). In 2010 Inoue et al.^2^ described POEM as a less invasive procedure to disrupt LES to improve food passage. It is myotomy of the circular esophageal muscle fibers endoscopically during a submucosal tunnel.

Although it is a fairly reliable and effective procedure, anaesthetic management of POEM introduces serious challenges for the anaesthesiologists.^3, 4, 5^ Carbon dioxide insufflation of esophagus combined with submucosal dissection and myotomy may lead to capnoperitoneum, pneumothorax, pneumomediastinum, subcutaneous emphysema, hypercarbia and increased airway pressure. Impaired esophageal emptying might result in aspiration of esophageal contents. Handling these problems is challenging in young children, especially in infants and requires experience. Current clinical recommendations are based on retrospective case series.^6, 7, 8, 9^ However, there are no case series involving pediatric patients. Therefore, we conducted this retrospective cohort study to investigate the perioperative complications and anaesthetic management of 16 pediatric patients with achalasia who underwent POEM.

## Methods

After approval from Research Ethics Committee of Koç University (approval no.: 2021.433.IRB1.125, date: November 26, 2021), records of patients under 18 years old who underwent POEM procedure were examined. Patient’s age, weight, body mass index (BMI) and hospitalization duration were obtained from patient charts. Peroperative monitoring and mechanical ventilation data was extracted from the anaesthesia records. These included heart rate, pulse oximetry, noninvasive blood pressure and end tidal carbon dioxide (ETCO_2_) measurements recorded at five minutes intervals and ventilation parameters, such as peak airway pressure (Ppeak), ventilation mode and respiration rate. Peak pressure and ETCO_2 _recorded in three instances as initial (in.), maximum (max.) and before extubation (end.) levels. Since it was a retrospective study, plato pressure, PEEP and compliance could not be accessed. Capnoperitoneum, pneumothorax, subcutaneous emphysema and intraabdominal needle decompression instances and other peroperative complications were also obtained from the anaesthesia recordings.

**Figure 1:**
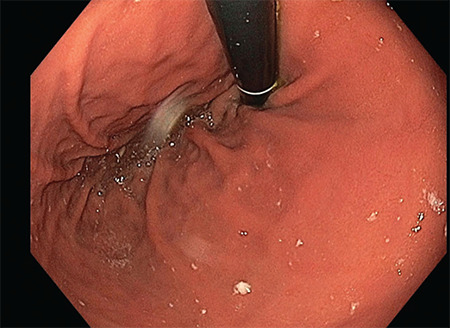
Endoscope, during the procedure

**Figure 2:**
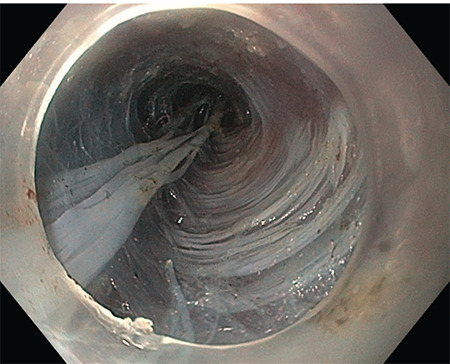
Submucosal tunnel, circular and longitudinal esophageal muscle fibers before myotomy

**Figure 3:**
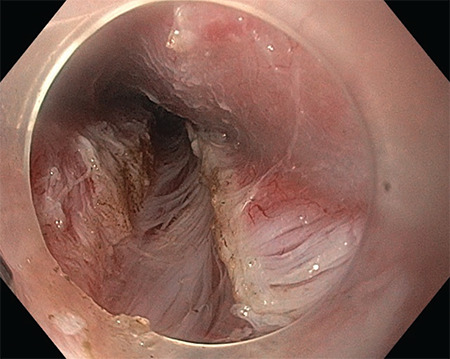
Longitudinal fibers and selective circular myotomy

It is performed endoscopically under general anaesthesia (Figure 1). During the procedure, a mucosal incision is made above LES. A submucosal tunnel is created starting at the incision site and ending below the gastroesophageal junction (Figure 2). Through the tunnel, myotomy of the circular esophageal muscle fibers are performed with an electrosurgical knife (Figure 3). Throughout the procedure, CO_2_ is insufflated through the endoscope.

### Preparation for POEM

Gastroscopy was performed in all the patients prior to POEM to exclude esophageal candidiasis and ulceration due to stasis related esophagitis. In subjects with esophageal candidiasis, oral antifungals were given for 2 weeks prior to the procedure.

According to instutional policy four days before the operation, patients were given a liquid diet with no particulates for 3 days. Twenty-four hours before the operation the diet was changed to a clear liquid diet and 6 hours before the operation all oral intake was stopped. No preprocedural esophagoscopy to empty the esophagus was performed.

Proton pump inhibitor therapy (pantoprazole; 0.5 mg kg^-1^ IV, twice daily) and prophylactic intravenous antibiotics (ampicillin/sulbactam or ciprofloxacin for those with penicillin allergy) were initiated before the procedure and continued throughout the hospital stay.

### Anaesthesia Induction and Maintenance

Patients were monitored with ECG, pulse oximetry, noninvasive blood pressure measurement initially and additionally with an ETCO_2_ monitor after intubation. Patients were positioned supine with the upper abdomen, thorax and neck exposed to facilitate observation of abdominal distension and subcutaneous emphysema.

Anaesthesia was initiated by rapid sequence induction (RSI) with propofol (2-3 mg kg^-1^), fentanyl (0.5-1 µg kg^-1^) and rocuronium (1 mg kg^-1^). Cricoid pressure was applied after the loss of consciousness until the cuff of the endotracheal tube was inflated. During the induction of anaesthesia, no regurgitation or aspiration was observed in any of the patients.

Anaesthesia maintenance was provided with 0.8-1.2 minimum alveolar concentration sevoflurane in a 40-60% O2-air mixture. Remifentanil infusion and bolus doses of rocuronium (0.1-0.2 mg kg^-1^) were administered throughout the procedure. Patients were ventilated in volume controlled ventilation mode (VCV) or pressure controlled ventilation mode (PCV) mode, depending on the attending anaesthesiologist’s preference. In PCV mode, inspirium pressures were adjusted when necessary to maintain adequate tidal volume. Respiration rate and minute ventilation were adjusted to maintain ETCO_2_ below 45 mmHg. There have been no hemodynamic changes requiring inotropic medication during either induction or maintenance.

Drugs and doses used for preoperative sedation, induction and maintenance of anaesthesia, perioperative analgesia and others were obtained. Narcotic analgesic doses were converted to morphine equivalents.

While POEM is generally considered safe and effective, there are certain patient groups for whom POEM might not be suitable in an endoscopy unit and who may require operating room conditions. Patients with complex anatomy or who have undergone prior surgeries in the upper gastrointestinal tract may have adhesions or altered anatomy that makes it difficult to perform POEM safely in an endoscopy unit. In such cases, the procedure may be better suited for an operating room where there is more space and equipment available. Patients with significant comorbidities such as severe cardiopulmonary disease or bleeding disorders may benefit from the controlled environment of an operating room where additional medical support and resources are readily available.

If a patient requires concurrent procedures along with POEM, such as laparoscopic fundoplication for reflux disease or a gastric procedure, performing these in an operating room may be more practical and efficient. Patients with high-risk features such as large esophageal diverticula or severe esophageal strictures may require additional interventions or monitoring that are more easily managed in an operating room environment.

Ultimately, the decision regarding the appropriate setting for performing POEM depends on various factors including patient-specific characteristics, procedural complexity, and available resources and expertise. The treating physician, in consultation with the patient and other healthcare providers, will determine the most suitable setting for performing POEM on a case-by-case basis.

### Management of Procedural Complications

Upper abdominal distension, elevated ETCO_2_ and Ppeak were initially managed by decompressing the stomach by suctioning with the endoscope. When this failed to lower the ETCO_2_ and/or Ppeak, and when significant upper abdominal distension was observed, needle decompression of the intraabdominal cavity was performed by the gastroenterologist. The median Ppeak value and peak airway pressure were recorded.

After the myotomy, paracetamol 10 mg kg^-1^ IV and narcotic analgesics were administered for analgesia. The mean narcotic analgesic dose converted to Morphine equivalent was 0.05 mg kg^-1^. Paracetamol 10 mg kg^-1^ four times each day was administered until the discharge from the hospital.

## Results

From June 2017 to September 2020, 16 patients under the age 18 underwent POEM procedure in our hospital. Median age was 5.5 with a range of 1-16 years (18-199 months) and 46% of the patients were males. Median BMI was 15.3 (12.5-27.1) kg m^-2^ 6 patients (40%) were ventilated in volume control mode and 10 (60%) in pressure control mode. Anaesthesiologists preferred PCV mode in younger patients. The median age was 3 (1-6) years for patients ventilated in PCV mode and 10 (3-16) years in VCV mode.

The median Ppeak value after the induction (inPpeak) was 24.5 cmH_2_O with a range of 15-34 cmH_2_O (Table 1). During the procedure Ppeak pressure of 11 patients were found higher than the Pin (69%).

ETCO_2 _increased in all cases. In 10 cases (62%) it was above 45 mmHg. The median increase was 7.5 mmHg with a range of 1-23 mmHg, which is difference between max and min ETCO_2_. The median of max ETCO_2_ was 45.5 (33-60) mmHg and the median of ΔETCO_2_ was 7.5 (1-23) mmHg. Table 2 shows the summary of ETCO_2_ levels among ventilation modes.

Capnoperitoneum was observed in all 16 patients as upper abdominal distention that persisted after suctioning of the stomach. Fourteen patients required needle decompression of the intraabdominal cavity due to a persistent increase of Ppeak or ETCO_2_ or observation of significant upper abdominal distension. Among these 14 needle decompressions, 9 of them were performed due to an increase of ETCO_2_, 1 due to the increase of Ppeak, 1 due to increases of both ETCO_2_ and Ppeak and 3 due to significant upper abdominal distension. Out of 10 patients who had elevated ETCO_2_ before the needle decompression, 6 had ETCO_2_ levels between 45 and 50 mmHg, 4 above 50 mmHg. ETCO_2_ decreased after the decompression in all of the cases with ETCO_2_ >50 mmHg, whereas in 3 (50%) cases with ETCO_2_ between 45 and 50 mmHg.

The only patient who required needle decompression solely for high Ppeak was decompressed when Ppeak was 35 cmH_2_O and remained at the same level after decompression. No complications related to needle decompression such as bleeding, bowel injury or peritonitis were observed in any of the patients.

Overall, 10 (71%) out of 14 decompressions were effective in improving the parameter that led to the need for decompression. Median ages of the patients with successful decompressions were lower than the ones with unsuccessful decompressions [49 (16-148) vs 182 (94-200) months respectively].

All patients had subcutaneous emphysema of the neck and upper thorax during the procedure. The diagnosis was performed by detection of crepitus and swelling of the neck and chest wall. No patient had emphysema below the chest or above the neck. In all cases emphysema was self-limiting and no complications such as dyspnea or hypoxemia were observed after extubation.

One patient has been inadvertently extubated during the procedure while removing the endoscope. The procedure was stopped and the patient was intubated again without any complications.

The mean procedure duration was 66.9 (22.9) minutes. The mean duration of anaesthesia from induction to extubation was 97.5 (31) minutes. All patients were extubated in the endoscopy unit without any emergence complications.

## Discussion

In our case series, regardless of ventilation strategies, ETCO_2_ values ​​increased during the procedure, due to mediastinal and peritoneal absorption, increasing the minute ventilation does not lower the ETCO_2_, if abdominal distension is observed, needle decompression of the intraabdominal cavity can be performed. The threshold of ETCO_2_ value was determined by the attending anaesthesiologist’s discretion. When ETCO_2_ >50 mmHg, 100% of needle decompressions lowered the ETCO_2_ whereas when ETCO_2_ was between 45 and 50 mmHg this ratio was 50%. Although this suggests that the threshold should be closer to 50 mmHg, further research is needed to determine the appropriate ETCO_2_ threshold in pediatric patients. However, complications like bleeding, bowel perforation and peritonitis should be taken into account. While there is no consensus on ETCO_2_ threshold for needle decompression in the literature, ETCO_2_ > 50 mmHg was proposed as a threshold in two studies on adults.^10, 11^ If the problem is not resolved with needle decompression, the procedure is stopped and the ETCO_2_ level is expected to return to normal.

The first concern in the anaesthetic management of patients undergoing POEM procedures is the risk of aspiration during the induction of anaesthesia. Some authors suggest preprocedural esophagoscopy to remove esophageal content.^3, 7, 8^ while others argue that this intervention itself carries its own risk of aspiration.^6, 9^ Esophagoscopic cleaning is especially important in patients with megaesophagus which is seen up to 10% of patients with disease duration longer than 10 years.^12, 13, 14, 15^ Since disease duration in pediatric patients is lower than in adults, megaesophagus is less likely to develop in children. Furthermore, preprocedural esophagoscopy is performed in awake or lightly sedated patients to minimize the risk of aspiration. This limits its use in pediatric patients.

As expected, none of the patients in this case series had megaesophagus and no preprocedural esophagoscopy was performed. RSI with cricoid pressure was used in all cases without any regurgitation or aspiration. However, in cases with megaesophagus, preprocedural esophagoscopy might be safer than RSI with cricoid pressure.

Pediatric patients may have smaller anatomy, which can make the procedure technically challenging and the esophageal wall in pediatric patients may be thinner and more delicate, increasing the risk of inadvertent perforation during the procedure. Pediatric patients may require specialized anaesthesia management due to their age and size, which adds complexity to the procedure. Children may have difficulty communicating symptoms postoperatively, making it challenging to assess their recovery and manage any complications.

Performing a POEM procedure on a pediatric patient in an endoscopy unit requires careful consideration of several factors, including anaesthesia. POEM procedures are typically performed under general anaesthesia to ensure the patient is unconscious and unable to feel pain during the procedure. It’s essential to have an anaesthesiologist experienced in pediatric anaesthesia present during the procedure. Pediatric patients have unique physiological and pharmacological considerations that require specialized expertise. Pediatric patients may require specialized airway management techniques, such as the use of smaller endotracheal tubes or supraglottic devices, to maintain a clear airway and adequate ventilation during anaesthesia. Continuous monitoring of vital signs, including heart rate, blood pressure, oxygen saturation, and end-tidal carbon dioxide, is essential throughout the procedure to ensure the patient’s safety. Adequate intravenous access should be established before the procedure to administer medications and fluids as needed during anaesthesia. A thorough preoperative evaluation of the patient’s medical history, physical examination, and laboratory tests should be conducted to assess the patient’s overall health and identify any potential risk factors. A tailored anaesthesia plan should be developed based on the patient’s age, weight, medical history, and the specific requirements of the POEM procedure. Adequate post-anaesthesia care should be provided to ensure the patient safely recovers from anaesthesia and any potential side effects or complications are promptly addressed. The endoscopy unit should be equipped with pediatric-sized equipment and supplies, including endoscopes, monitors, and anaesthesia delivery devices, to accommodate the needs of pediatric patients. Collaboration between gastroenterologists, anaesthesiologists, nurses, and other healthcare professionals is essential to ensure the safe and successful performance of POEM procedures in pediatric patients.

In total, there were 14 needle decompressions performed which correspond to 87.5% of the patients in this case series. In the previous 3 studies comprising a total of 739 adult patients, needle decompressions were performed in 126 (17.1%) of patients.^13^ Higher incidence in our study might be due to a lower threshold of ETCO_2_ used in 6 of the cases. However, the remaining indications for decompression (ETCO_2_ > 50 mmHg, Ppeak ≥ 35 cmH_2_O and significant upper abdominal distension) still amounts to 8 (50%) of the cases. This difference between adults and children is most likely due to the thinner muscle barrier in the esophageal wall leading to easier diffusion and escape of CO_2_ to the mediastinum and intraabdominal cavity.

In all previous studies on anaesthesia of POEM, inferences were made on the ETCO_2 _monitoring and complications in adult patients. In this study, better information was gained about ETCO_2 _monitoring and complications in pediatric cases.

## Conclusion

During the POEM procedure, end-tidal CO_2_ rises in all cases and needle decompression of abdominal cavity might be required. ETCO_2 _and Ppeak thresholds for needle decompressions need to be determined in further studies.

## Ethics

**Ethics Committee Approval:** This study approved by Research Ethics Committee of Koç University (approval no.: 2021.433.IRB1.125, date: November 26, 2021).

**Informed Consent:** Retrospective study.

## Figures and Tables

**Table 1. Perioperative Airway Pressures During POEM Procedures:** 

**Mode**	**inP_peak_**	**maxP_peak_**	**∆P_peak_**
PCV	22.5 (15-28)	27 (18-40)	4.5 (2-12)
VCV	25.5 (17-34)	26 (17-35)	0.5 (0-3)

inP_peak_, peak airway pressure after induction of anaesthesia; maxP_peak_, maximum peak airway pressure during the procedure; ΔP_peak_, maxP_peak_ - inP_peak;_ all units are in cmH_2_O, data represented as median (range).

POEM, peroral endoscopic myotomy; PCV, pressure controlled ventilation mode; VCV, volume controlled ventilation mode.

**Table 2. Perioperative ETCO2 Levels and Ventilation Modes:** 

**Mode**	**inETCO_2_**	**maxETCO_2_**	**ΔETCO_2_**
PCV	38.5 (35-50)	47.5 (40-60)	9 (1-22)
VCV	33 (31-45)	45.5 (33-55)	7 (1-23)

inETCO_2_, ETCO_2_ after induction of anaesthesia; maxETCO_2_, maximum ETCO_2_ during the procedure; ΔETCO_2_, maxETCO_2_ - inETCO_2_, all units are in mmHg, data represented as median (range).

ETCO_2_, end tidal carbon dioxide; PCV, pressure controlled ventilation mode; VCV, volume controlled ventilation mode.
